# Use of Online Health Forums by People Living With Breast Cancer During the COVID-19 Pandemic: Thematic Analysis

**DOI:** 10.2196/42783

**Published:** 2023-02-07

**Authors:** Sally Sanger, Suzanne Duffin, Rosemarie E Gough, Peter A Bath

**Affiliations:** 1 Information School, Faculty of Social Sciences University of Sheffield Sheffield United Kingdom; 2 School of Health and Related Research Faculty of Medicine, Dentistry and Health University of Sheffield Sheffield United Kingdom

**Keywords:** online health forum, breast cancer, COVID-19, pandemic, discussion forum, coronavirus, web-based communities, information use

## Abstract

**Background:**

At the time of the UK COVID-19 lockdowns, online health forums (OHFs) were one of the relatively few remaining accessible sources of peer support for people living with breast cancer. Cancer services were heavily affected by the pandemic in many ways, including the closure of many of the customary support services. Previous studies indicate that loneliness, anxiety, distress, and depression caused by COVID-19 were common among people living with breast cancer, and this suggests that the role of OHFs in providing users with support, information, and empathy could have been of increased importance at that time.

**Objective:**

This study aimed to examine how people living with breast cancer shared information, experiences, and emotions in an OHF during the COVID-19 pandemic.

**Methods:**

This qualitative study thematically analyzed posts from the discussion forums of an OHF provided by the UK charity, Breast Cancer Now. We selected 1053 posts from the time of 2 UK lockdowns: March 16, 2020, to June 15, 2020 (lockdown 1), and January 6, 2021, to March 8, 2021 (lockdown 3), for analysis, from 2 of the forum’s boards (for recently diagnosed people and for those undergoing chemotherapy). We analyzed the data using the original 6 steps for thematic analysis by Braun and Clarke but by following a *codebook* approach. Descriptive statistics for posts were also derived.

**Results:**

We found that COVID-19 amplified the forum’s value to its users. As patients with cancer, participants were in a situation that was “bad enough already,” and the COVID-19 pandemic heightened this difficult situation. The forum’s value, which was already high for the information and peer support it provided, increased because COVID-19 caused some special information needs that forum users were uniquely well placed to fulfill as people experiencing the combined effects of having breast cancer during the pandemic. The forum also met the emotional needs generated by the COVID-19 pandemic and was valued as a place where loneliness during the pandemic may be relieved and users’ spirits lifted in a variety of ways specific to this period. We found some differences in use between the 2 periods and the 2 boards—most noticeable was the great fear and anxiety expressed at the beginning of lockdown 1. Both the beginning and end of lockdown periods were particularly difficult for participants, with the ends seen as potentially increasing isolation.

**Conclusions:**

The forums were an important source of support and information to their users, with their value increasing during the lockdowns for a variety of reasons. Our findings will be helpful to organizations offering OHFs and to health care workers advising people living with breast cancer about sources of support.

## Introduction

### Background—COVID-19 and Breast Cancer

COVID-19 had an enormous impact worldwide, as countries struggled to contain and manage the disease. A strategy to manage the spread of COVID-19 was to implement lockdowns at the local, regional, and national levels during the pandemic. During the UK lockdown periods, people other than essential key workers were required to stay at home, except for undertaking essential shopping and physical exercise. Health services for non–COVID-19 conditions were severely reduced, and some health care professionals were reallocated to care for patients with COVID-19. Many support services were also closed. However, one of the few relatively unaffected sources of support for patients was online health forums (OHFs). These are sections of websites where groups of people facing similar health-related issues, life challenges, or difficult circumstances can communicate and provide each other with emotional support; advice; and information, especially experiential information. OHFs are distinct from chat rooms in that they are asynchronous, whereas chat rooms facilitate discussions that occur in real time.

Patients with cancer were greatly affected during the COVID-19 pandemic [1,2]. People with cancer had high risks of COVID-19 (breast cancer was deemed a *moderate risk*) [2-5] and patients undergoing chemotherapy had increased mortality risk from COVID-19 [3,4] because of immunosuppression. Patients experienced delays, cancellations, and alterations both to treatment and their overall care in a time of rapid change and great uncertainty [5].

Interaction with other people with the same disease or condition, going through the same experiences, can be highly beneficial to patients [6-8]. Peer support services can normally be undertaken face-to-face; however, during COVID-19 lockdown periods, this was not possible. Therefore, OHFs became an increasingly important opportunity for patients for support [9].

Web-based options can provide benefits similar to face-to-face interactions [10] and have additional benefits; for example, use is not limited geographically, participants can meet 24/7 [11,12], and there is great anonymity. During the COVID-19 pandemic, women in a breast cancer online support group reported numerous advantages over meeting in person [12], for example, not having to wear a wig and being able to accommodate the meetings with family responsibilities.

### Literature Review

#### Cancer During the COVID-19 Pandemic

As noted previously, COVID-19 had major effects on cancer services and treatment including delays or cancellations in diagnostic and treatment services and restricted access to health services, including screening [2,13-16]. Some patients were also reluctant to visit health care settings [2,17] owing to the increased risk of infection [2,14,15].

COVID-19 also affected patient experience and well-being, including restricting access to psychosocial support from personal or professional networks [16], limiting support during hospital or office visits, and limiting communication with medical personnel. Patients with cancer reported receiving misinformation or insufficient information about COVID-19 during the pandemic [18]. Some also experienced increased levels of isolation and loneliness [18-20], fear, and anxiety [17] and mental health issues [21,22]. However, other studies found that some patients reported being less stressed, lonely, and unhappy than carers of patients with cancer or people without cancer [17,23]. Some patients felt reduced isolation and increased sense of being part of society again, as everyone was at home [24-26].

#### Breast Cancer During the COVID-19 Pandemic

People living with breast cancer experienced many of the abovementioned negative problems throughout the cancer journey [27-29]. Although many patients were “treated according to pre–COVID-19 guidelines” [30], other studies [19,31] predicted additional avoidable deaths, even up to year 5 following diagnosis.

Increased levels of loneliness and high levels of anxiety, depression, and psychological disorder caused by COVID-19 stressors were reported among people living with breast cancer [32-37]. Their quality of life was affected according to their level of concern about COVID-19 [38], and high levels of fear of cancer recurrence were also associated with COVID-19–related anxiety and distress [39]. Patients also experienced concerns about the risk of COVID-19 when immunosuppressed and distress caused by attending treatment alone, and some patients experienced a great burden of responsibilities at home (eg, because of homeschooling) [40].

#### OHFs During the COVID-19 Pandemic

Dedicated OHFs were set up during the pandemic, specifically to discuss COVID-19. The most popular topics discussed were symptoms, public health practice, and psychological impacts [41]. Participants mostly provided feedback or opinion, and the most frequently used sources of information were the news and websites.

Discussion about COVID-19 was also found on other OHFs and social media. Users who had great web-based support had better subjective well-being, better mental health, and more prosocial behaviors [42], which led to a sense of belonging and reduced loneliness. A US survey of 28 OHFs for people with chronic illnesses over 13 months found that they were used more during peak lockdown times and the desire for emotional or mental health support increased over time. When moderated well, OHFs can “provide a powerful, intermediate and safe space where conversations about mental and emotional wellbeing can be normalized” [43]. However, potential negative effects include the distress of hearing about others’ negative experiences regarding COVID-19 [44].

#### Cancer-Specific and Breast Cancer–Specific OHFs During the COVID-19 Pandemic

Cancer OHFs are an effective way of sharing and receiving information and social and emotional support [45-47]. A Cochrane review of online support groups for women with breast cancer did not find studies of sufficient size or quality to determine evidence of improvement or lack of improvement to negative affect (such as anxiety, depression, or distress), through use of the groups. However, it noted the benefits that were clear to women participating in online support groups [47].

Lung and breast cancer were frequently discussed on Twitter and OHFs by people living with cancer during COVID-19 lockdowns, with concerns regarding “delayed diagnosis, cancellations, missed treatments, and weakened immunity” [48]. Forum users were worried about the impact of COVID-19 on treatment, health, everyday life, and finances—they expressed a strong need for information and advice on COVID-19 and self-management [49]. The emotional health of patients with breast cancer was affected by COVID-19, and they desired one-to-one therapies, advice, and emotional support [50]. Social distancing led many patients “to turn to online forums for support” [9].

#### Comparison With Previous Literature

The literature shows that conversations about the pandemic were not confined to forums specifically dedicated to COVID-19 and that it was deemed a topic suitable for forums for people living with breast cancer. Although the COVID-19–related topics discussed in the forums and the emotions that COVID-19 caused have received research attention, there are only a few studies focusing on what the breast cancer forums were used for and how they were used. Previous studies have not examined in depth how people living with breast cancer shared information, experiences, and emotions in an OHF during the pandemic. Zhang et al [9] conducted qualitative analysis of posts from a US-based forum for people living with breast cancer, focusing only on how the users felt about the treatment delays and their views of treatment, rather than on the role of the forum more generally. Green et al [43] investigated the users of 28 OHFs with varied chronic health conditions at different stages of the COVID-19 pandemic. They compared user responses to a survey over a few days from a selection of months between March 2020 and April 2021, rather than covering 2 lockdown periods as done in this study. Their study was US-oriented, was non–cancer-specific, did not look at data from the forums during these times, and focused on information sources about COVID-19 rather than information needs more broadly. Loeb et al [51] conducted a study of posts on prostate cancer forums, which compared posts from before the pandemic with those at its beginning, not during 2 lockdown periods, as in this study. It is also important to consider how people at different stages of the cancer journey used OHFs during the pandemic, and no previous studies have specifically explored this. Similarly, no previous study has explored how people at different stages of the breast cancer journey may have used OHFs during the COVID-19 pandemic. Therefore, the overall aim of this study was to address these research gaps by undertaking an in-depth qualitative analysis of posts made by people living with breast cancer during the COVID-19 pandemic and, through this, to better understand how people living with breast cancer used OHFs during the COVID-19 pandemic.

#### Research Questions

The following research questions (RQs) are addressed in the paper:

- RQ1—How did people living with breast cancer share information, experiences, and emotions in an OHF during the COVID-19 pandemic?
- RQ2—How did people’s use of OHFs differ during different lockdown periods during the pandemic?
- RQ3—How did people living with breast cancer at different stages of their cancer journey share information, experiences, and emotions during the pandemic?

## Methods

### Research Design and Approach

This qualitative study explored the use of an OHF for people living with breast cancer during the COVID-19 pandemic. It continues the work of *A Shared Space and a Space for Sharing* project [45,52]. We thematically analyzed posts from an OHF provided by the UK charity, Breast Cancer Now (BCN). This approach enabled us to observe forum use discussion that was uninfluenced by the presence of a researcher and foregrounded the participants’ own comments on COVID-19 and its impact on them. In addition, we obtained some basic descriptive statistics to provide some information about the forum users.

### Study Setting

Breast cancer is the most common form of cancer in the United Kingdom and can have a wide range of outcomes. For some, it may form an acute individual episode; for some, it becomes a chronic disease that they live with for many years; and for others, the prognosis is terminal. BCN is a charity providing support, information, advice, and help to people living with breast cancer at all stages of their journey. We chose BCN because it is publicly accessible; allows its forum data to be used for research; and had previously been involved in *A Shared Space and a Space for Sharing* project, and thus, a well-established working relationship was already present before this study. The discussion forum part of the site offers 12 boards covering different aspects or stages of breast cancer, for example, *local recurrence or new primary diagnosis*, *top tips and practical support*, and *radiotherapy*.

### Sampling Strategy

#### Forum Boards

We reviewed all 12 boards and identified the most relevant ones. Searches were then conducted on these using key COVID-19–related terms (eg, “Coronavirus,” “COVID-19,” and “Lockdown”) to identify the boards that contained the most discussion about the pandemic. The findings were discussed with BCN, and the boards, *recently diagnosed with breast cancer* (RDwBC) and *chemotherapy monthly threads* (CMTs), which contained a large amount of material on COVID-19, were selected for analysis and to answer RQ3. CMTs are (sometimes very long-lasting) threads designed for all those starting chemotherapy in a particular month. They support small cohorts of individuals experiencing the same process together over a period. In addition, chemotherapy is a highly stressful time involving long hospital visits and very high risks of infection and therefore a time when COVID-19 was likely to be of much concern. We chose the RDwBC board because it was likely to reflect the concerns of individuals immediately following their diagnosis and at the beginning of their treatment, a period that is very stressful and typically involves a high level of contact with health professionals and medical appointments. COVID-19 was likely to be a significant concern for the individuals posting on this board, at a time when they needed considerable support.

#### Forum Threads and Posts

To answer RQ2, we purposively focused on 2 UK lockdown periods: March 16, 2020, to June 15, 2020 (lockdown 1), and January 6, 2021, to March 8, 2021 (lockdown 3). A short second lockdown in November 2020 was not included in this study. March 16, 2020, was selected because this was when cancellations of appointments began and the UK government instructed “now is the time for everyone to stop non-essential contact and travel” [53]. June 15, 2020, was when nonessential shops reopened in the United Kingdom. January 6, 2021, was the official beginning of the third lockdown, and March 8, 2021, was when restrictions started to lift and schools began to reopen.

We selected 6 CMTs initiated during the relevant time period (March-June 2020) and the previous month and 2 other short threads with titles explicitly about COVID-19–related matters that fell within the time period. For lockdown 3, a total of 7 CMTs that began during the relevant time period (January-March 2021) and the previous month were selected. There were no threads with titles explicitly about COVID-19–related matters during this period.

The final sample of 15 threads was downloaded, pseudonymized, and identifiable material such as photos was removed. Then, we loaded the data into NVivo (version 12; QSR International) and conducted inductive and deductive semantic and latent thematic analyses. Posts referring to COVID-19 and its impact on users or the forum were coded. Comments on the value of the forum that did not specifically relate to COVID-19 were also coded to provide a more complete picture of why the users valued the forum at the time.

We followed the same process for the RDwBC threads, with some modifications. The number of posts per thread was found to be much lower than that for CMTs; therefore, we included threads that started before the chosen time period but contained posts within it. The time period was also extended to the end of June 2021, which was the point at which full unlocking occurred in the United Kingdom for lockdown 3. In total, 35 threads were identified for lockdown 1 and 48 threads for lockdown 3.

### Data Analysis

#### Qualitative Analysis

We analyzed the data using the original 6 steps for thematic analysis by Braun and Clarke [54] but by following a *codebook* approach [55]. This started with in-depth familiarization with and reflection on the data. The team agreed on deductive codes based on the initial readings and then manually coded the data sets in NVivo (version 12) with the unit of coding being the whole post. Codes were also added inductively and reflexively throughout. Codes were regularly discussed and preliminary themes were generated through ongoing team discussions.

To help identify areas of analysis where different understandings could be explored and which could benefit from deep reflection or reflexivity on our part, a coding comparison test was conducted on a subset of posts using the coding comparison query function in NVivo. In total, 50 randomly selected posts from each of the 2 data sets were coded independently by 3 members of the research team using NVivo. The coding comparison query was run among the researchers. Coding consistency was high, with approximately 80% agreement. The exercise was primarily valuable in generating discussions about codes and development of themes. Then, we recoded all data using the final set of agreed codes and checked data extracts to ensure that they were coded appropriately. The final thematic map was developed through discussion within the entire research team.

#### Quantitative Analysis

Quantitative analysis was performed using data extracted from the posts from both lockdowns in both CMT and RDwBC data sets. We extracted the date, time, user pseudonym, and user type from each post to analyze the number of users and posts and their distribution across the lockdown periods. Posts that had been coded during qualitative analysis and were therefore deemed COVID-19–related were extracted, transformed, and loaded into MATLAB (MathWorks), and descriptive statistics were derived using MATLAB.

### Ethics Approval

Ethics approval was obtained from the University of Sheffield (application 043811) in January 2022. We obtained permission from BCN to access the data, consistent with good practice [56].

### Confidentiality

We pseudonymized the data after downloading them, by replacing usernames with pseudonyms and removing identifying details, such as names and photos. Quotations from the forums were carefully altered to protect participants’ identities, while ensuring that the post’s meaning was retained [57]. Idiosyncratic or rare words and spellings, which were likely to be found easily owing to their infrequent occurrence, were changed to more common alternatives with the closest possible meaning.

## Results

### Background—COVID-19 as a Topic of Conversation

#### Quantitative Data

We found that COVID-19, especially in relation to cancer, was a frequent topic of conversation during both lockdown periods. It clearly interested group users, and the forum was considered as an appropriate venue for discussion. To understand the context of use of the forum for COVID-19 as a topic of discussion, we looked at the number of users and posts and their distribution across the lockdown periods, as described in the *Methods* section.

In total, at least 1053 posts were related to COVID-19 across the RDwBC threads and CMTs during lockdowns 1 and 3. Of these 1053 posts, 719 (68.28%) were in the CMTs and 334 (31.72%) were in the RDwBC threads. There were some differences in the frequency of posts between the RDwBC and CMTs during the 2 time periods. In the RDwBC thread, the number of posts was higher during lockdown 1 (196/334, 58.7%) than during lockdown 3 (138/334, 41.3%), whereas the CMTs were more evenly split across lockdown 1 (381/719, 52.9%) and lockdown 3 (338/719, 47%). This may be owing to the reduced immunity of those undergoing chemotherapy, which meant that COVID-19 remained as a matter of great interest to them for longer than it did among people coping with a recent diagnosis in 2021 who were not immunosuppressed.

The distribution of COVID-19–related posts across lockdowns 1 and 3 was analyzed to determine if there were changes in the frequency of posting across lockdown periods. In the CMT during lockdown 1, there was increased activity at both the beginning (March 15, 2020, to April 10, 2020) and the end (May 10, 2020, to June 14, 2020) of the lockdown period. However, during lockdown 3, activity did not increase at the beginning of the lockdown, but a large spike of activity occurred toward the end (February 15, 2021, to March 9, 2021). The RDwBC threads had no initial spikes in activity; instead, during lockdown 1, there was increased activity in the middle period (April 16, 2020, to May 16, 2020), and there were no clear spikes during lockdown 3. This highlights how users used the forum for both immediate information around COVID-19 and continued to use the forum for information throughout the lockdowns.

Finally, we analyzed individual forum users who posted across threads and lockdowns. Although there were more posts in the CMT (719/1053, 68.28%), there were only 80 different users compared with the RDwBC group (334/1053, 31.72% of posts), which had 106 different users. To identify frequent users, searches were performed for people posting ≥20 posts on a thread. There was a high number of such users in the CMTs (16/80, 20% of users) compared with those in the RDwBC threads (5/106, 4.7% of users). These frequent users accounted for 43.97% (463/1053) of the total posts around COVID-19, suggesting that there are groups of superusers who post frequently. However, a substantial number of users were discussing about COVID-19, highlighting the importance of the forum for many users.

#### Qualitative Data

Specific aspects of COVID-19 and breast cancer that were discussed in the forums included the following:

1. The impact of COVID-19 on the treatment and experience of cancer, for example, changes, cancellations, or delays to treatments and having no visitors during inpatient care
2. The impact on cancer services, including professional support services, such as those offered by the UK charity, Macmillan Cancer Support
3. The increased risk that COVID-19 posed to patients with cancer, especially those undergoing chemotherapy
4. COVID-19’s impact on the patient’s support network of family, friends, and neighbors, both in terms of how they supported the patient and the effect on their own lives
5. COVID-19’s impact on the forum users’ daily life including work; education; and social activities, for example, holidays, exercise, and shopping
6. COVID-19–specific topics. Forum users exchanged information and advice on topics ranging from shielding to booking vaccinations, from dealing with COVID-19–related anxiety to where to get items in short supply because of the pandemic. They asked questions that they may not have wanted to bother their oncology team with, such as the following example:

Anyone had the jab already? Wondering where they inject it...Just asking cos I have a line in one arm and my surgery scar on the other.

Ethel; lockdown 3

This illustrated a practical problem that patients with cancer could face regarding COVID-19 because of the nature of cancer treatment. Another example is the timing of receiving the vaccine in relation to the chemotherapy cycle. These forum conversations provide an interesting picture of the impact of COVID-19 on the life of people with breast cancer.

We observed that the people posting in the forums quickly adopted terms and expressions that had come to have COVID-19 associations in UK society in general. This includes COVID-19–specific terms such as “LFT” (lateral flow tests), phrases such as “we’re all in it together” and being on the “frontline” (signifying National Health Service [NHS] COVID-19 wards), and commenting on living in “strange,” “difficult,” or “scary” times. There were references to the “current state of the world” or “current climate” used as euphemisms for the pandemic, and the injunction to “stay safe” was a recurrent slogan, frequently used in the forums as it was in wide conversation, especially during the first lockdown.

### Themes

#### Overview

We identified four main themes from the analyses under the overarching first theme of *COVID-19 amplifies the forum’s value to users*:

1. Using the forum to meet special information needs
2. Using the forum to share emotions generated by cancer during COVID-19
3. Using the forum to reduce isolation
4. Using the forum to raise each other’s spirits

The following sections discuss the themes individually.

#### Theme 1—COVID-19 Amplifies the Forum’s Value to Users

Although the forum was highly valued by its users in ordinary times for providing access to others who understood them (because they had been through the same experiences), this value acquired an extra dimension in the lockdown periods. To grasp this fully, it is necessary to appreciate the unique nature of the users’ situation at that time—they were the first people in UK history to be diagnosed with cancer or undergoing chemotherapy during a nationwide lockdown caused by a pandemic. People who had had breast cancer before could, and did, still offer peer support that was appreciated. For example, several forum users commented very positively on the BCN *someone like me* service, which matched people to trained volunteers with similar experiences. However, no previous patients with cancer had experienced cancer at the same time as a global pandemic causing lockdowns. Their unique circumstances led to some special information needs (refer to theme 2) and situation-specific emotions (refer to theme 3) in the context of which the forum acquired additional value. In the forums, people living with breast cancer could connect with others in this unique position, who fully understood this:

This is my first post. I was recommended this forum as a way to connect with others going through such a scary experience at such a crazy time.

Tina; lockdown 1

This was particularly the case during lockdown 1—people who were diagnosed or treated at that time formed a small group that could support those during lockdown 3:

You’ve found the right place to be with people who understand. I was in my fifties and single when I was diagnosed in early 2020 and went through treatment in the pandemic, you can get through it too.

Donna; lockdown 3

During the lockdowns, there was a lack of alternative ways to connect with peers because of COVID-19. Most customary sources of peer and professional support and information about cancer were either closed, difficult to access, or reduced in quality:

It’s been very hard that so many support services have had to close. This forum has been a lifeline for me. So a huge thank you. It is so good to be able to connect with people going through this.

Joanne; lockdown 1

I was fortunate I got to go on a...course before the pandemic and lockdown...Now they can only offer online courses.

Gretchen; lockdown 1

Personal support networks, for example, friends and family, were also difficult to access other than via the web:

A diagnosis of breast cancer is hard enough in “normal” times, but SO much harder for anyone during COVID-19, who can’t easily and physically access their network of family and friends for much needed support.

Cheryl; lockdown 3

Therefore, the forum’s value was amplified as a rare source of peer support and information. Its value was further increased as it was available 24/7, which made it more accessible than peer support meetings that had moved online:

I took part in an online support group this morning...recommended if you like that kind of thing - although, it’s only once a month which isn’t enough for me...here we have our wonderful little team of lovely women.

Maeve; lockdown 3

The forum was also a place where members could support others, even if COVID-19 meant that they could not help at work. There were several mentions of guilt from key workers who were unable to help their colleagues “on the frontline”:

I totally understand how you feel about this horrendous situation we are in. I also work for the NHS and my team are working so hard helping as many patients as they can. I wish I could be well enough to help.

Philippa; lockdown 1

Another individual had replied by saying the following:

All you people in the NHS are amazing - even though you can’t be there, please know that you’re certainly helping here.

Megan; lockdown 1

Therefore, the forum acquired additional value to its participants above and beyond the already high value in which it was held, as it gave continuous access to others going through the unprecedented situation of living with breast cancer during the COVID-19 pandemic.

#### Theme 2—Using the Forum to Meet Special Information Needs

During lockdowns 1 and 3, when discussing about COVID-19, users used the forum in 2 main ways: first, to recount or compare their experiences or both, and second, to ask questions and exchange information and advice. These often went together because forum members used their experiences to answer questions, pooling their knowledge to create a store of information. These were not novel ways of using the forum, as discussions around breast cancer were also largely in this format. However, what was different was that these periods were times of particular uncertainty around information, with UK Government and NHS guidance about COVID-19 and cancer changing rapidly. There were also geographical variations across the UK nations and regions and NHS Trusts, which complicated what information was valid where. Information at these times was confusing or absent:

When you’re self-isolating it’s hard to know what’s best to do - advice from the government keeps changing.

Roberta; lockdown 1

Rebecca - I hope you get your appointment. I think hospital policies are changing daily. Hopefully things will continue for now.

Nancy; lockdown 1

As before the pandemic, forum users frequently referred each other for information to their NHS breast care team members, especially the nurses [49]. The resulting experiences were mixed, suggesting that COVID-19 changes may have rendered acquiring information from these sources difficult for some people:

I found having this during COVID-19 has been hard as in hospital everyone has to keep their distance and many of my appointments have to be over the phone so it feels like there’s less support and it’s hard to ask all those questions over the phone.

Lauren; lockdown 1

My Breast Cancer Nurse got transferred to the coronavirus team early on too.

Joan; lockdown 1

The forum was needed as a source of information more than ever:

I know just how you feel as I’ve been booked in for my surgery with very little information or preparation. However, everyone here has been so helpful, it’s made all the difference to me and to my family who have been reading some posts here too.

Rowan; lockdown 1

Is it alright for me to still come here as this is the only place I am getting answers to my questions?

Stephanie; lockdown 3

Forum users shared practical tips and ideas about COVID-19 and cancer. They shared resources, for example, websites for homeschooling, information about support services that were still open, and numbers to access priority shopping slots. During lockdown 3, they helped each other with booking vaccinations, including managing information systems to get a response. Responding to Tara, who described her frustrating attempts over several days to book, “Alexa” offered a tip that had worked for her:

Check out all the NHS sites...One of them asks if you are happy with the info...I clicked “no” and that sent me right to the actual booking site.

Alexa; lockdown 3

This did not work for “Tara,” and “Alexa” then asked a family member to help:

The great thing about this forum is that somebody somewhere always has an answer.

Alexa; lockdown 3

This is very practical informational support based on experience that would not be available through more conventional sources. The forum provided them with a wide range of people to consult with during the COVID-19 pandemic. It became a place where they could ask “Is it like this where you are?” and “Has anyone else been told this – is it normal?”:

My partner rang oncology and was told that my consultant has changed my treatment...He asked why and it’s so I’m not there as long! I don’t understand, it’s only going to slightly reduce the time I’m there. If it makes no difference why were we to have it in the first place? Feel very confused...has anyone else been told this?

Philippa; lockdown 1

Through this, they could also check rumors and share potentially useful news about COVID-19:

Really anxious with this virus and what it means for future treatments. I’ve heard some have been cancelled, and that the cold cap option isn’t available, is this right? Or does it vary by area? Also heard can’t have anyone with us while getting chemo. All speculation for me right now.

Rebecca; lockdown 1

When I went last week they said they are planning to send some NHS patients to private chemo units to keep some NHS space free so I don’t think they’re planning to stop chemo.

Joanne; lockdown 1

Comparing experiences in the forums and finding what was happening elsewhere, at a time of confusing and conflicting information, gave them useful information and a route to some sense of normality and reduced isolation.

The author, Case [58], identified 2 types of information needs: objective needs, which relate to facts and are driven by rational judgment, and subjective needs, which arise from a need to make sense of the world and are driven by feelings and emotions [58]. During the COVID-19 pandemic, the forums showed both types of needs being expressed, sometimes intermixed. For example, “Diane” introduced herself as “feeling pretty low at the minute! and confused, angry etc etc.” She described her medical situation and the difference in her experience with NHS cancer services at a recent appointment because of COVID-19. She concluded by saying the following:

I feel so lost and isolated. My nurse did not ring. this did not surprise me as I had been in the breast unit, it was empty, normally bursting at the seams! and this virus! I just do not know what to expect. I was told that if the cancer spread to my lymph nodes treatments meant I had a good chance of 5 years.

Diane; lockdown 1

She mixed specific questions with the need to make sense of her situation (feeling lost). In return, she received much encouragement and accounts of other people’s experiences, and she stated the following:

Thank you for the responses. I will ring my key worker tomorrow and see if there is an update. It’s really good hearing positive outcomes.

Diane; lockdown 1

Other forum users had provided specific advice to help answer her question, but they had also addressed her more general anxiety through accounts of their experiences.

#### Theme 3—Using the Forum to Share Emotions Generated by Cancer During the COVID-19 Pandemic

As is customary with OHFs, the forum was a place where members could share their emotions and support each other. The overriding emotions related to living with breast cancer during lockdown 1 of the COVID-19 pandemic were anxiety and fear, felt most frequently at the beginning of the pandemic in March 2020:

These are frightening times so not surprising you are anxious about going for treatment and scared of catching something. I think it’s totally normal to be worried about it all. We should be, as we have to be careful.

Joanne; lockdown 1

My anxiety levels are sky-high and I hate to say it but I feel like the hospital has abandoned me a little [I feel very guilty for even thinking that at this difficult time]. I feel lost and absolutely terrified about what the next few months will bring.

Sara; lockdown 1

The anxiety expressed on the forum at this stage was largely related to the risk of infection, impact that COVID-19 would have on treatment, possibility of delays or cancellations, and other general worries about COVID-19. A conflict between the desire for treatment and the fear of infection was common:

We have to self-isolate but at the same time we have to go to hospital for treatment. Aaaargh!

Joanne; lockdown 1

My hospital has just been identified as one of the biggest COVID hot spots in the country. Although I desperately want to proceed with treatment I feel very frightened.

Roberta; lockdown 1

In the RDwBC threads, anxiety was primarily voiced about changes to treatments because of COVID-19, and there was great uncertainty among users, as they did not yet have a definite plan. In CMTs, anxiety was primarily shared around vulnerability to SARS-CoV-2 infections. This is likely to reflect that people posting in the RDwBC threads had not yet started treatment, whereas people posting in CMTs had started and had reduced immunity.

Sharing feelings of anxiety reduced during lockdown 1 and had greatly reduced by lockdown 3 (although it remained as the most commonly expressed emotion). During lockdown 3, COVID-19 was no longer a terrifying unknown, and help was on the horizon in the form of vaccines. The cause of the shared anxiety shifted to include new reasons, for example, the lifting of lockdown and the resulting increased likelihood of coming into contact with others:

I completely agree with you about the lockdown easing. I am terrified, haven’t been anywhere for months...The thought of sitting next to people on any kind of transport, - ick!

Alexa; lockdown 3

During lockdown 3, within the CMTs, mentions of stress were approximately as prevalent as comments on anxiety.

The forum was a place where users could share these emotions with people other than loved ones who could be affected by their feelings. They could vent; complain; and receive reassurance, empathy, and comfort:

I wanted to try and share my experiences so far to give some reassurance or comfort that you will be well looked after. Yes this is scary, particularly so right now with everything else going on, but we’re in this together and we can share our journeys here and support each other.

Greta; lockdown 1

Reassurance came to the fore in comments from users about the forum’s value when they specifically connected this to COVID-19. It was less prevalent in general comments about the usefulness of the forum, with no reference to COVID-19.

COVID-19 also caused some distinctive emotions specific to the time, which group members used the forums to cope with. This was presumably because other members were uniquely placed to understand these, whereas outsiders and even family members may be negatively affected. The first could be summed up as “it’s bad enough having cancer without COVID-19 as well!” (or the converse). This situation was frequently acknowledged empathetically (“it’s not surprising you are anxious with COVID-19 as well”) or to explain the extreme anxiety and fear experienced:

This is a really difficult time isn’t it. I feel that the pandemic situation has me constantly on high alert, which makes it even harder to deal with the natural anxieties to do with treatment.

Joan; lockdown 1

I just wanted to say I feel exactly the same, I start my chemo very soon and like you I feel frightened and so anxious, while this lockdown makes it so much worse.

Edie; lockdown 1

Sometimes, there was also a sense of unfairness, unreality, and almost disbelief regarding the extraordinary circumstances they found themselves in:

I’m feeling your worries too, it’s like we’ve all been handed one of the biggest battles to deal with, and they pile the end of the world on top.

Megan; lockdown 1

This was especially common during lockdown 3, when exasperation, empathy, and sympathy were evident, perhaps because COVID-19 had become annoying and worrying:

Totally get where you are coming from. As if having breast cancer isn’t bad enough we all have to go through it during a pandemic! Terrible timing.

Letitia; lockdown 3

I can’t believe you’re having so much hassle with the jab!!! You’ll get there but you SO don’t need that right now.

Maeve; lockdown 3

A second distinctive emotion was annoyance or being upset at people without breast cancer complaining about COVID-19 when they did not have breast cancer:

It does irritate a bit when people complain about having to stay in or not taking this seriously. Grr! I suspect we’ll be doing this for a lot longer than most so we have more to complain about!

Yvonne; lockdown 1

My parent friends don’t really understand why I’m being so careful or how dangerous it could be if I sent my child back to school and they brought the virus home.

Gretchen; lockdown 1

The forum is the perfect place to offload this feeling without giving offense or causing distress. Lou et al [59] also noted that “participants receiving active treatment reported...greater concern that the general public does not adequately understand the seriousness of COVID-19.”

Thirdly, there was emotional upset at COVID-19 “eclipsing” cancer, potentially reducing its seriousness and importance to others:

For a few days last week I felt like the cancer had been eclipsed by coronavirus which was really upsetting but then I became pro-active in reaching out...

Ruth; lockdown 1

Some members simply did not reach for support outside the forum because of COVID-19:

I’ve not felt able to tell my parents yet. They’ve been ill, they’re shielding because of the virus and I know they will struggle to cope with the news and knowing that they can’t do anything to help.

Gina; lockdown 1

...It’s a strange time, with others suffering with COVID-19 to the point that I feel guilty talking about my cancer.

Erica; lockdown 1

People with breast cancer may have deferred going to their physician or had tests delayed because of COVID-19:

I found a lump just before lockdown. Waited a couple of weeks due to the madness that was happening with COVID-19.

Noreen; lockdown 1

I’ve convinced myself that it’s going to be advanced cancer and that it’s spreading massively every day I am waiting for surgery. All made worse [because] my routine mammogram was delayed for several months because of COVID-19.

Trisha; lockdown 3

Interestingly, COVID-19 was seen by some as providing others with an excuse for not being supportive or understanding. The following comment was about health care professionals:

Some people I click with, some I feel wary of or they seem to not be bothered. The pandemic seems to help them not bother too much either.

Bethany; lockdown 1

I do wonder if some hide behind the mask and COVID-19 is an excuse...

Alexa; lockdown 3

Thus, the forum was a place where emotions could be expressed and empathy received without stressing loved ones and with the assurance that the forum user would be understood. For the emotions that were specific to cancer during COVID-19, there were very few other places to discuss this.

#### Theme 4—Using the Forum to Reduce Isolation

Isolation is an important theme when discussing cancer OHFs in general, as many users come to them because they feel alone with their condition and want to meet someone in the same situation. For those receiving chemotherapy, shielding from contact with others may have been necessary to avoid infection, even without the pandemic [39]. However, clearly, lockdowns seriously exacerbated the isolation of many individuals:

Cancer feels lonely however much support you have as it’s so very difficult for others to understand what it is like, and even more difficult with all this chaos around.

Iris; lockdown 1

As noted in theme 1, COVID-19 removed or altered access to both professional and personal support networks. Close contact (including hugs) was not advised, and cancer services (eg, consultations) moved to the web, which suited some people but not others. There was an added dimension of isolation in that people (even if present) could not be seen well because of masks and other personal protective equipment (PPE; eg, “It’s really strange when you can’t see people’s faces because of masks. It does make me feel even more alone”) Social distancing sometimes made it difficult to talk to fellow patients, and no visitors were allowed in wards or at home. As far as possible, people were kept away from health care settings to reduce infection risk.

People who lived alone became particularly isolated because of lockdowns:

I do live on my own, which is usually fine, but with COVID-19 it’s pretty isolated as I can’t see people properly...Couple of doorway visits...I do miss visits and people coming to cheer me up.

Maeve; lockdown 3

As noted in theme 1, the forum’s value was increased because of this situation:

It’s all so strange right now not being able to see anyone – I’m so grateful for this forum and all of you here.

Andrea; lockdown 1

I think that while we are isolating this [forum] will be more helpful than ever.

Nancy; lockdown 1

However, interestingly, lockdown could also help with isolation in some ways, as everyone was in the same situation. The person living with breast cancer was not missing out on anything, as they would be under normal circumstances:

At least for those of us currently going through chemo – the rest of society is joining us now in lockdown so we are not missing out on much.

Zoe; lockdown 1

For others, lockdown could be the opposite of isolation, as family members may have been around all the time. This could be annoying, for example, interfering with work or requiring demanding homeschooling of children. Visits for cancer treatments or tests even became a source of relief:

Having first round of chemo in a few days...several hours with no company...not sure if that sounds dreadful or wonderful after being stuck in the house with hubby and toddler lol!!

Chris; lockdown 1

By lockdown 3, comments on excitement over mundane errands had become a recurrently shared joke:

Just had my best day in months...guess what I did...I LEFT MY HOUSE AND WENT TO THE GARDEN CENTRE!! WHOOP-WHOOP!! It was fantastic!

Maeve; lockdown 3

Heading out to get my picc line flushed today. I’m getting a trip out! Yay!

Amanda; lockdown 3

When lockdowns lifted, the sense of isolation could intensify, with users having mixed feelings about this:

I know it’s mean but this isolation felt better because everybody had to do it. Now I reckon I will feel a bit jealous if restrictions are lifted. I feel bad for feeling this way.

Francesca; lockdown 1

We are all going through such a hard time, lockdown in a very selfish way has helped massively. No one can go anywhere right now so bad though it is, I dread the unlock when I will still have to remain inside.

Alexa; lockdown 3

It seems plausible that this sentiment would be difficult to share outside the group. It suggests that users may have extra need for forum support and the understanding of others in the same situation at the end of lockdowns, similar to the beginning.

#### Theme 5—Using the Forum to Raise Each Other’s Spirits

Members used the forum to lift each other’s spirits in various ways specific to the time.

First, they identified and shared other positive aspects of having cancer during the COVID-19 pandemic, in addition to those described previously. The most commonly mentioned aspect in the CMTs was that there was no need to worry about losing hair as no one would be able to see it:

One positive thing - there’s no one to see my ever increasing baldness.

Henrietta; lockdown 1

Other positive aspects that were shared on the forum to raise spirits included the following:

1. Few demands on people as they were not having to go to work
2. Being able to save money for postcancer or post-COVID-19 treats
3. Coming alone to chemotherapy meant that they could meet other patients and talk to the staff (if social distancing rules permitted)
4. Having more time with the family could be enjoyable (and this also meant that there were people at home to look after the person living with breast cancer)
5. Working from home and homeschooling had their positive aspects, including providing distraction from thinking about cancer

In RDwBC, there were fewer examples of forum users describing the positives of cancer during the COVID-19 pandemic than in CMT. This may be because potential positives were overshadowed by the shock of diagnosis and concerns about when treatment would start.

Second, as noted in the CMTs, users shared small pleasures that had replaced large ones during the COVID-19 pandemic:

I never thought I’d find a trip to [supermarket] something to look forward to so much! Lol!

Chris; lockdown 1

Third, throughout the pandemic, and particularly during lockdown 3, forum users also used the forum to daydream and plan what they would do after cancer and COVID-19:

One day soon we’ll be able to enjoy those glasses of wine with our friends again and all this will be over! Xx

Leah; lockdown 3

Those good times are ahead of us, we just have to be very patient and enjoy dreaming about it in the meantime xx

Maude; lockdown 3

Finally, they also raised one another’s spirits through humor, which was seen as an important function of the forum:

My bins have been out more than me...I might start calling myself “Dusty,” I might have more chance of getting out...and seeing “men”!!!! or even just people or another human being without full PPE on.

Alexa; lockdown 3

The forum clearly played an important role in raising spirits and allowed expressions of humor, which outsiders may not have fully appreciated.

## Discussion

### Summary

This study has developed a better understanding of how people living with breast cancer used the BCN OHF during the COVID-19 pandemic. The study shows that people living with breast cancer found the OHF to be a helpful form of support for sharing and discussing information, experiences, and emotions about COVID-19 and related topics during the pandemic (RQ1). The topics discussed during the 2 lockdown periods we studied had much in common; however, some differences in forum use were found, for example, regarding the topics discussed and levels of emotions experienced (RQ2). Some differences in use depending on the forum user’s stage of their journey were found; however, overall, the similarities were more striking, suggesting common interests regarding COVID-19 throughout the pandemic (RQ3).

### Principal Findings

We found that group members frequently used their forum (RQ1) to talk about COVID-19, particularly its impact on the experience of cancer, its treatments and services, and its effect on social activities and support. This focus was practical and personal—there was little theoretical discussion about the pandemic. For example, in the threads studied, there were no discussions about the latest statistics (other than a passing mention that they were “scary”) and any of the government’s approaches or how COVID-19 developed and spread. There was little discourse on COVID-19 symptoms, unlike forums dedicated to the pandemic [40]. During lockdown 3, there was only passing mentions about conspiracy theorists, such as “anti-vaxxers” and COVID-19 deniers. This suggests that users saw this forum as a place for practical help around COVID-19 rather than for theoretical discussion. This may be because they were accustomed to using it for practical help with cancer.

Forum users found additional value in sharing information, experiences, and emotions in the OHF (RQ1) for various reasons. The pandemic exaggerated emotions and situations that may be occurring anyway because of breast cancer, for example, anxiety, fear, and loneliness. Participants were in a situation that was “bad enough already” and COVID-19 amplified this. In addition, COVID-19 created some special information needs and emotions that its users were uniquely well placed to fulfill. It was also valued as a place where loneliness may be relieved and spirits lifted in ways specific to the time. Overall, COVID-19 amplified the value of the forum to its users.

*Isolation* was found to be an important theme, both in the physical, literal sense of being alone and the emotional sense of feeling lonely and as a synonym for *shielding*. Cancer is described as a lonely experience, because those who have not had it do not know what it is like. Even fewer people understood what it was like to have breast cancer during the COVID-19 pandemic. The value of the forum increased as it was one of the few remaining options for communication and support when other sources were missing or limited, for example, owing to social distancing or PPE. However, group users’ views of the isolation caused by COVID-19 were nuanced—in some ways, it helped them to feel better about the cancer experience as they were not missing out, with everyone being in the same situation [19,25].

The 2 lockdown periods studied had much in common, but some differences in use were found regarding the topics discussed and levels of emotions (RQ2). During lockdown 1, COVID-19 was unknown, with no good treatments and no vaccine; therefore, it is unsurprising that accounts of fear and anxiety were more prevalent. During lockdown 3, these were reduced—by then, COVID-19 was more familiar and less threatening.

Both the beginning and end of the lockdown periods had particular challenges that the forums could be used to express. There were several unknowns and uncertainties at the beginning of both lockdowns (during lockdown 1, COVID-19 was completely new, but even during lockdown 3, eligibility for early vaccination was a new issue). The end of lockdowns potentially increased the sense of isolation, as patients saw everyone else resuming normal life, whereas they had to remain cautious. Although in the future, there may be few, if any, full lockdowns, COVID-19 remains as a great problem for patients with cancer, especially those undergoing chemotherapy, than for those without cancer. This means that some of the questions and issues raised, especially around shielding-related matters, will still be relevant to patients today, particularly with episodic surges in COVID-19 cases, and potentially for any future pandemics.

Overall, some differences in use were observed between the 2 boards studied (RDwBC and CMT), and it was shown that there are plausible reasons why we may expect this (RQ3). However, the degree of commonality was more striking. This suggests that the findings may also be transferable to other boards within the forum; however, this would require further studies to test this.

### Comparison With Previous Studies

This study contributes to the limited body of work using OHF post analyses to illuminate aspects of cancer during the COVID-19 pandemic [1,9,48]. It confirms these works’ findings about the topics related to COVID-19 (such as treatment delays and changes to the cancer experience) that are discussed in cancer OHFs. It also supports their findings about the dominant negative emotions (notably fear and anxiety) that COVID-19 engendered in group users. However, this study differed in several ways from the other studies. For example, this study covered a longer period of the first lockdown than the study by Colomer-Lahiguera et al [1], and this study compared it with the third lockdown and thus was able to comment on the changes in topics between the 2 periods.

Colomer-Lahiguera et al [1] focused on describing people’s experiences of cancer and did not examine COVID-19’s effect on the way the forum was used. It analyzed only 230 posts and selected only the first post of each thread, rather than analyzing ongoing discussions during the period. Moraliyage et al [48] also analyzed the topics discussed rather than the use of the forum, as did Zhang et al [9]. Loeb et al [51] compared posts on a prostate cancer forum during and before the pandemic, found low rate of misinformation (7%), and again focused on topics of concern. This study is the first to focus on the impact of COVID-19 on the use of a breast cancer OHF.

Hulbert-Williams et al [16] noted the positive aspects of COVID-19, as did Kassianos et al [23] and Schellekens and van der Lee [24]. This study extends the understanding of the ways in which forums contributed to positive well-being, through its analysis of the different ways in which people used the forum to lift each other’s spirits.

Our study supports that of Patel et al [60] and Zhang et al [9], who noted that forums were used the most at the beginning of the first lockdown. It also supports the finding by Green et al [43] that interest in COVID-19 had decreased substantially by later lockdowns. There was less discussion about it by RDwBC users, and there were no threads specifically for COVID-19 in the CMT forum. Changes in the topics discussed between the periods were noted.

Finally, this study contributes to the debate about the value of self-help groups to users, as seen, for example, in the editorial by Cordero [44], which asked whether such groups were “a necessity or an added calamity” during the COVID-19 pandemic. Cordero [44] focused on groups dedicated to COVID-19, particularly groups in the Philippines. We would echo his suggestions for user protection on the web, but dispute the predominantly negative assessment, at least in the UK context. This study clearly shows that the value of the forum increased for BCN users as a consequence of the way in which COVID-19 amplified existing information needs, negative emotions, and sense of isolation.

### Strengths and Limitations

The study analyzed users’ own conversations, which were unaffected by the researchers. The conclusions are based on users’ interests and perspectives and thus indicate their priorities and interests as expressed during peer support. The study also benefited from the ongoing collaboration and discussions with the forum provider, BCN.

This study was limited in only focusing on 1 cancer OHF and 2 stages of the cancer journey. It did not include other BCN threads, for example, those for radiotherapy or other treatments, secondary diagnosis, or end of life. The fact that there was much commonality across the 2 stages analyzed may suggest that similar issues may be found in other stages; however, it is also plausible that there would be additional issues and differences.

The findings presented in this paper are not intended to be generalizable to specific groups but may be transferable to other online support groups during the COVID-19 pandemic, particularly other cancer and breast cancer forums.

### Recommendations for Breast and Other Cancer OHFs for Similar Situations

Some of the recommendations in this section apply if COVID-19 escalates again or if other similar health emergencies or pandemics arise, necessitating lockdowns. Other recommendations are for the groups regarding the continuing impact of COVID-19.

#### Support During Societal Transition Points

There was increased anxiety and fear at the beginning of lockdowns; therefore, particular attention from moderators (or their equivalents) is warranted at that point, with provision of reassurance. Groups also need to be aware that users may feel great anxiety and isolation at the point when lockdowns lift. This is not intuitive; therefore, it is important that cancer support services are aware of this and offer extra support, if possible. Even when services are moving offline again, the question remains whether people living with breast cancer will feel safe to take them up or prefer to use the web. Therefore, groups may want to consider their short-term service plans.

#### Providing Other Ways to Connect With Peers

Groups are in a unique position in facilitating access to multiple others going through cancer during lockdown. Therefore, they may like to investigate other ways to facilitate connection among these peers, for example, live chat rooms. The users spoke highly of BCN’s *someone like me* service, and it would be useful for other groups to consider developing a similar service.

#### Special Services for Individuals Living Alone

Those living alone may be at particular risk of loneliness, and groups may consider whether they would benefit from a specific board or a thread for them; however, people living alone are particularly vulnerable and additional precautions should be considered.

#### Raising Awareness of How COVID-19 Can Eclipse Cancer

There may be a need to raise awareness among cancer professionals that “COVID-19 eclipsing cancer” can be a cause of distress. Previous literature has shown that it can lead to actions that are not in the patients’ best interest, for example, staff or patients delaying diagnostic tests or treatments. However, this study demonstrates that it can also extend to users hiding cancer from their personal support networks of family and friends, thus becoming very isolated.

### Future Studies

The transferability of our findings to other breast cancer groups or breast cancer sections on general cancer websites should be explored. Identifying differences from other site-specific cancer OHFs would be valuable to health care professionals, patients, and researchers.

Studies have shown that people also benefit from *lurking* in OHFs—only reading posts, rather than posting themselves. Future studies could examine whether the number of *lurkers* on the BCN forums increased during the lockdowns. This would indicate another population that may have turned to forums owing to the lack of other sources and whose interests require exploration; however, identifying and recruiting people to such a study could be challenging.

This study does not explore whether there was anything that reduced the usefulness of the forum or acted as a barrier to its use during the lockdowns. Interviews with *posters* and *lurkers* could explore whether there were issues that they felt they could not talk about in the forums regarding cancer during COVID-19, and, if so, where they went to discuss those issues. It could explore whether, for example, they ever felt that there were topics they could not raise on the web or that they had to word certain topics more carefully. This would be very useful to OHFs supporting people with breast cancer.

Finally, according to Dhada et al [2], “...the evidence base relating to caregivers (about COVID-19 and cancer) is limited, with only two studies reporting their perspectives. Further research in this key population is warranted.”

Given that caregivers of people living with breast cancer found COVID-19 to be very difficult [16,23], future studies are needed to explore their use of BCN or other forums during the COVID-19 pandemic.

### Conclusions

As well as the COVID-19 pandemic continuing, there may be similar pandemics in the future, and people living with breast cancer remain very vulnerable. Although, for the rest of society, life may return to normal following COVID-19 and future pandemics, people living with breast cancer remain at great risk. The evolving nature of global pandemics, as we have learned from the COVID-19 pandemic, means that there are times of greater risk than during the periods we studied; for example, tests are no longer free, and legal requirements for people with COVID-19 to self-isolate are removed. Under these circumstances, there are more opportunities to come in contact with the virus, and there may be less support for shielding people. Moreover, waiting lists for cancer diagnosis and treatment remain affected by COVID-19, with much longer waiting times than before the pandemic. In short, people living with breast cancer are still likely to be experiencing a high level of concern about COVID-19 and the accompanying anxiety and distress. Therefore, OHFs are an important source of support and information for their users, both during the COVID-19 pandemic and future pandemics.

## Abbreviations

BCN: Breast Cancer Now
CMT: chemotherapy monthly thread
NHS: National Health Service
OHF: online health forum
PPE: personal protective equipment
RDwBC: recently diagnosed with breast cancer
RQ: research question

## Data Availability

All data generated within the study were derived from the Breast Cancer Now online health forum, and, similar to previous studies [45,52], we will not be able to provide access to these data ourselves. Breast Cancer Now should be contacted directly to request access to their data.
